# Natural Pigments of Bacterial Origin and Their Possible Biomedical Applications

**DOI:** 10.3390/microorganisms9040739

**Published:** 2021-04-01

**Authors:** Rodrigo Salazar Celedón, Leticia Barrientos Díaz

**Affiliations:** 1 Laboratory of Molecular Applied Biology, Center of Excellence in Translational Medicine, Universidad de La Frontera, Temuco 4810296, Chile; r.salazar04@ufromail.cl; 2 Scientific and Technological Bioresource Nucleus (BIOREN), Universidad de La Frontera, Temuco 4811230, Chile

**Keywords:** biomedicine, bacterial pigments, biomolecules

## Abstract

Microorganisms are considered one of the most promising niches for prospecting, production, and application of bioactive compounds of biotechnological interest. Among them, bacteria offer certain distinctive advantages due to their short life cycle, their low sensitivity to seasonal and climatic changes, their easy scaling as well as their ability to produce pigments of various colors and shades. Natural pigments have attracted the attention of industry due to an increasing interest in the generation of new products harmless to humans and nature. This is because pigments of artificial origin used in industry can have various deleterious effects. On this basis, bacterial pigments promise to be an attractive niche of new biotechnological applications, from functional food production to the generation of new drugs and biomedical therapies. This review endeavors to establish the beneficial properties of several relevant pigments of bacterial origin and their relation to applications in the biomedical area.

## 1. Introduction

**Figure 1:**
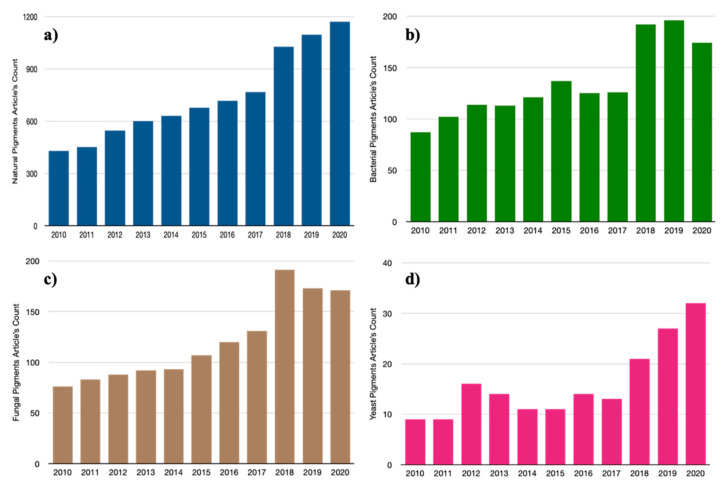
The articles indexed in NCBI database related to (**a**) “Natural pigments”; (**b**) “Natural Pigments and Bacteria”; (**c**) “Natural Pigments and Fungi”; (**d**) “Natural Pigments and Yeast” trends in the last 10 years.

Natural products were the beginning and main source of treatments for hundreds of years; however, the application of bioactive natural metabolites in traditional medicine, and the discovery of new drugs continue to be active and constant [1]. Microorganisms are an abundant source of novel bioactive compounds since, unlike higher organisms, they are a source of easily renewable resources that give rise to production with a potentially greater yield [2]. Among the bioactive compounds of microbial origin, natural pigments have attracted the attention of industry due to an increasing interest in the generation of new safe, easily degradable, ecologically friendly products with no adverse effects [3]. Actually, the amount of “natural pigment” published articles doubled during the last 10 years, which microorganisms like bacteria, yeasts, and fungi contributed to the increased amount of research articles through these years (Figure 1) [4]. This may be attributed to the wide range of pigments and dyes of artificial origin being used in the production and manufacture of foods, dyes, cosmetics, and drugs, which have had various adverse effects [5]. One of the main consequences of these synthetic additives is cell damage due to oxidation, which can lead to immunosuppression in the human being which, in the worst-case scenario, can involve carcinogenesis [6]. Pigments of natural origin play an important role in the physiology and molecular processes of microorganisms because they act as a method of adaptation to various extreme environments, have a protective function against solar radiation, and are also involved in functional processes like photosynthesis [7]. Additionally, being influenced by environmental factors, microorganisms give rise to a variety of pigments with unique characteristics mainly related to the connection between the microorganism and the ecosystem. At the moment, an extraordinary range of microbial pigments in various environments has been described, such as carotenoids, flavins, phenazines, quinones, monascines, violaceins, indigoidines, melanins, among others [3,8,9]. Several studies grant these natural pigments a variety of beneficial properties such as anticancer activity, pro-vitamin A, and desirable characteristics such as high photostability, thermal stability, pH, and a great contribution to the preservation of biodiversity while reducing the release of harmful chemicals into the environment as a result of the manufacture of synthetic dyes [10]. Of all the microorganisms capable of producing natural pigments, bacteria, yeasts, and fungi are the most relevant in the area. In the case of fungi, multiple pigments have been described and have been used for years as taxonomic identifiers, and some of them are commercially available for cell staining and protein detection as well as being used as replacements for the production of synthetic dyes in the textile industry [11,12,13]. Yeasts stand out for the production of yellow, orange, and reddish pigments, which are used in the wine and aquaculture industries, as well as in medicine [14,15,16,17,18]. On the other hand, bacteria offer certain distinctive advantages over their counterparts, due to their short life cycle, their low sensitivity to seasonal and climatic changes, their easy scaling as well as their capacity to produce pigments of various colors and shades [7]. On this basis, bacterial pigments promise to be an attractive niche of new biotechnological applications that can be mass-produced quickly and aimed at several industrial areas, from functional food production to the generation of new drugs and biomedical therapies. This review endeavors to establish the beneficial properties of several relevant pigments of bacterial origin to then relate them to possible applications in the biomedical area and for human health.

## 2. Natural Pigments of Bacterial Origin

Among bacteria, pigment production is highly variable, although usually present in Actinobacteria. Several genera, such as *Streptomyces*, *Nocardia*, *Thermomonospora*, *Microbispora*, *Streptosporangium*, *Rhodococcus,* and *Kitasatospora* produce a wide variety of pigments [19]. While the main application of these pigments is in the textile [20], food, and cosmetic industries [21], pigments like melanin, quinones, violaceins, and indigoidines have been reported as good antimicrobial agents [10]. Besides, pigments can be used as antioxidants, bioindicators, and anticancer agents [22]; thus, their potential is becoming an important field of biomedical application. Of the entire range of bacterial pigments available, those described next have the qualities necessary to be used in the biomedical industry as promising therapeutic agents (Table 1).

## 3. Violaceins

**Figure 2:**
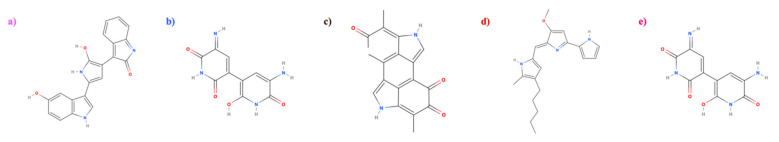
Molecular structures of the following bacterial pigments (**a**) Violacein; (**b**) Indigoidine; (**c**) Melanin; (**d**) Prodigiosin and (**e**) Rhodopsin. (Structures made using MolView software) [74].

Violacein, a purple dye (Figure 2a), is a natural compound of indolocarbazole formed by the condensation of two tryptophan molecules [23]. Its molecular mass is 343,341 g/mol and it is insoluble in water, soluble in alcohols such as methanol, ethanol, and acetone, soluble in DMSO, methanol, and ethyl acetate, and slightly soluble in dioxane and acetone [24,25]. All the species known to be able to produce violaceins are heterotrophic, have predominantly been discovered in aquatic ecosystems and many representatives of these species have been isolated from cold environments, such as Arctic ice, freshwater, and marine habitats [26]. The initial activity reports on this pigment date from 1942, indicating that raw extracts of a purple pigment produced by the strain *Chromobacterium violaceum* mixed with bacterial suspensions prevented the soil amoebas from ingesting these bacteria, which would otherwise be phagocytized [27]. In general, *Chromobacterium violaceum* is the most studied bacterium in terms of violacein production. However, this pigment has also been described in the genera *Janthinobacterium*, *Alteromonas*, *Collimonas*, *Duganella*, *Pseudoalteromonas,* and *Iodobacter* [23,25,26,28]. The biosynthesis and biological properties of this pigment have been studied extensively, and its production in nature, exclusively intracellular, may be related to a defense mechanism against fast-growing organisms by detaching vesicles loaded with this molecule, which can destroy nearby membranes [26]. This was verified recently when this compound was related to a lethal effect in *Batrachochytrium dendrobatidis*, an amphibian-specific fungus [28]. Finally, multiple biotechnological applications have been attributed to this pigment (Table 1), acting as an antibacterial, antiparasitic, antiviral, antitumor, and has genotoxic activity in cell lines [24]. In the last five years, five new strains have been identified as producers of this pigment; these belong to the genera *Pseudoalteromonas*, *Iodobacter*, *Janthinobacterium,* and *Massilia.* This last one has never before been described as a violacein producer [23,26,28]. On the other hand, the anticancer properties of the purple pigment have been evaluated, attributing it to the ability to induce extrinsic and intrinsic mechanisms of apoptosis in MCF-7 breast cancer cells [29]. This was observed in a similar assay performed by Ferreira and his collaborators [30], where violacein was proposed as a therapeutic agent for the treatment of leukemia, demonstrating the range of possible applications for this bacterial pigment.

## 4. Indigoidines

Indigoidine is a natural pigment formed by the condensation of two L-glutamine molecules catalyzed by a non-ribosomal peptide synthetase (NRPS). It is characterized by a very distinctive blue coloration, similar to indigo, from which it gets its name (Figure 2b). This compound is triggered in bacteria by previously known biosynthetic gene clusters. The biosynthesis of this pigment is similar in different bacterial strains; however, the genes charged with the biosynthesis of indigoidines are often repressed or silenced, which carries with it the reduction or null synthesis of this compound [31,32]. Multiple bacteria capable of producing this pigment have been described; initially, it was discovered in the 1960s as being produced by the strain *Pseudomonas indigofera* and later found in genera such as *Arthrobacter*, *Erwinia*, *Corynebacterium*, *Clavibacter*, *Vogesella*, *Phaeobacter*, *Photorhabdus*, *Dickeya,* and *Streptomyces* [32,33,34,35]. To date, although there has been an extensive characterization of the synthesis and structures of the pigment, its biological activity is still little known. It is estimated that indigoidine provides the strain that produces it with an increase in resistance to oxidative stress, acting as an antioxidant, protecting the bacterium from reactive oxygen species [7,34]. Also, there are reports that indigoidine has antibacterial activity against *Vibrio fischeri* (Table 1), inhibiting its growth [36]. Actual studies have concentrated mainly on understanding the pigment’s usefulness and function. It is currently noted that indigoidines have antibacterial activity against *Vibrio fischeri* [37]. However, experimenting to establish a greater range of antibiotic activity range in light of a larger number of bacterial species becomes imperative. In the last few years, Day and his collaborators [33] found that the production of indigoidine by bacteria can provide the microorganism with a competitive advantage in the environment. In this context, different physiological functions for indigoidine have been proposed, including as antioxidant molecules that help protect the cells against oxygen radicals, as possible intracellular signaling molecules that contribute to the modulation of swimming motility, among other cellular processes, or as antibacterial agents that suppress the colonization of competing organisms in the environment.

## 5. Melanins

Melanin is a heterogenous and polymeric pigment produced by eukaryote organisms and several microorganisms through fermentative oxidation [38]. In microorganisms, melanin is a molecule of high molecular weight and is particularly complex from an analytical point of view, since it is very stable photochemically and practically insoluble in most organic dissolvents, acids, and water [39]. Melanins can be classified into two large types: dark brown eumelanin and yellow or reddish pheomelanin, where the main difference between the two is the absence (eu) or presence (pheo) of sulfur in the composition of the pigment [42]. The basic structural unit of melanins is usually represented by covalently bound indolic compounds (Figure 2c), and most melanins seem to be indol-based polymeric compounds that contain variable amounts of other pre-indolic products [43]. In addition to being a biopolymer derived from natural sources, melanin has shown good biocompatibility and biostability, demonstrating an absence of side effects related to cytotoxicity and the antigenic response in living organisms, which is complemented by a relatively high half-life due to the absence of enzymes that can degrade this class of pigments in living cells [42]. Different bacterial melanins have been described in different species, such as *Rhizobium* sp., *Bacillus thuringiensis*, *Pseudomonas aeruginosa*, *Klebsiella* sp. and *Modestobacter versicolor* [44]. We know that the genus *Streptomyces* is widely studied as a source of antimicrobials and secondary metabolites, and 80% of known antibiotics come from *Streptomyces* [45]. A key method for identifying this genus is melanin-type pigment production [46]. Melanin has a photoprotective role against radiation in organisms and cells, protecting them from the radiation coming from the UV regions of the visible spectrum (Table 1), and being able to then sequester reactive oxygen species, reducing the damage from UV radiation [47]. Besides, due to its chemical composition, melanin has physicochemical properties that allow it to act not only as an ultraviolet ray absorber but also as a cation exchanger, drug carrier, semiconductor, X-ray, and gamma-ray absorber [48]. Melanins have been widely described mainly for their photoprotective effects in living organisms with a view to biomedical applications. However, their polymorphic properties make their study a constant challenge [49]. In recent years, new melanin-producing strains have been described with various biological effects, with antioxidant, anticancer, photostable, and heat-stable capacities [40,48]. Besides, studies have begun on prototypes of melanin nanoparticles for the treatment of diseases as well as the use of this pigment in chemotherapy and photothermal therapies [41,42]. In this context, melanin molecules of natural origin are excellent candidates with unique properties for biomedical and biotechnological applications [42].

## 6. Carotenoids

**Figure 3:**
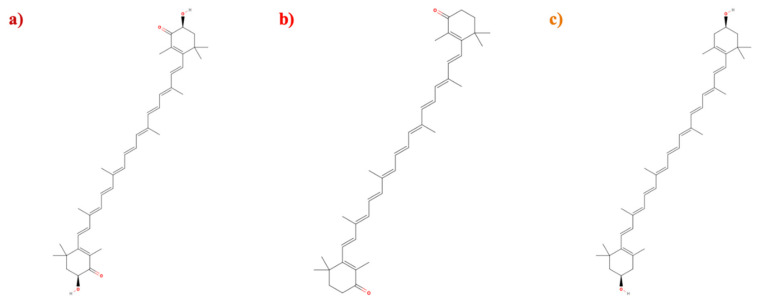
Molecular structures of the following bacterial pigments from the carotenoid family (**a**) Astaxanthin; (**b**) Canthaxanthin; (**c**) Zeaxanthin. (Structures made using MolView software) [74]).

Carotenoids are the most widely distributed pigment class in nature, showing yellow, red, and orange colors [50]. They are formed by the condensation of isoprenyl units (Figure 3) and are produced by a variety of organisms, from non-phototrophic prokaryotes to higher plants [51]. They are essentially hydrophobic molecules typically associated with photosynthetic membranes. It is believed that they do not move freely within the lipid interior of these membranes, but rather they are non-covalently bound to specific pigment-protein complexes [52]. Carotenoids are generally classified into two groups: carotenes (α-carotenes and β-carotenes) and xanthophylls (zeaxanthin, canthaxanthin, and astaxanthin) [53]. In industry, carotenes have garnered attention mainly due to their beneficial effects on health (Table 1), being able to increase the immune response, being involved in the prevention of cancer, and having high antioxidant properties [50]. The consumption of diets rich in carotenoids strengthens the immune system and reduces the risk of degenerative diseases such as cancer, cardiovascular diseases, macular degeneration, and cataracts [53]. Currently, carotenoids are highly biotechnologically significant due to their antioxidant capacities, being used as nutritional supplements as well as in cosmetic formulations [54]. More than 700 different types of carotenoids have been reported, with β-carotene, astaxanthin, canthaxanthin, lutein, lycopene, and zeaxanthins being the most important and of the greatest industrial value [53]. Due to their wide distribution, carotenoids are ubiquitous for a variety of microorganisms. In bacteria, these pigments play a role in adapting to different environmental conditions, and it is possible to find them in areas of high chemical radiation and areas with low temperatures and high ultraviolet radiation [9,51]. Considering the vast number of existing carotenoids, their multiple applications, and relevance to health, carotenoids are currently considered a growing niche in new biotechnological applications. The most noteworthy subclasses in terms of their biomedical applications are astaxanthins, canthaxanthins, and zeaxanthins. An astaxanthin that originated from the strain *Pontibacter korlensis* AG6 has been shown to have antibacterial, antioxidant, and cytotoxic properties in breast cancer cell lines [55]. New methodologies of metabolic engineering are being evaluated to increase the production of astaxanthins, canthaxanthins, and zeaxanthins using bacterial strains [56,57,58].

## 7. Prodigiosins

Prodigiosin is one of the most common and recognizable natural products, due mainly to its vibrant shiny red belonging to the family of prodiginines. This was first characterized from *Serratia marcescens*, a Gram-negative bacterium belonging to the family Enterobacteriaceae, ubiquitous and prone to producing a variety of pigmented colonies that often contain prodigiosin [5,59,60]. The biosynthesis of this pigment is based on the coupling of two precursors (2-octenal and proline), which form the linear tripyrrole (Figure 2d) that gives the reddish pigmentation during the stationary phase of bacterial growth [60,61]. In addition to *S. marcescens*, it is possible to find prodigiosin to a lesser extent in other genera such as *Janthinobacterium*, *Streptomyces*, *Vibrio*, *Hahella*, *Zooshikella,* and *Pseudoalteromonas* [59]. Prodigiosin is one of the most promising pigments from a biomedical point of view, because it has a variety of pharmaceutical properties (Table 1), acting as an antimicrobial (against foodborne pathogens), algicide, anti-inflammatory, anticancer, antimalarial, antidiabetic, as well as being an immune system modulator [6]. Some of these activities have already been described; for example, it can induce apoptosis in cancer cells, alter mitochondrial function and oxidative phosphorylation, interrupting, also, pH regulation when sequestering protons, hindering electron transport chains [62]. It has been demonstrated that prodigiosin inhibits the growth of a wide range of Gram-positive bacteria, including *Bacillus subtilis* and *Staphylococcus aureus*, as well as Gram-negative bacteria such as *Escherichia coli*, *Salmonella enterica,* and *Erwinia carotovora* [63]. However, despite decades of research, the mechanisms of antibacterial activity of this pigment remain poorly described. Without a doubt, the production of this pigment will continue intriguing microbiologists, clinicians, and bioprocess engineers [5]. It has been possible to clarify its method of action of being present as an antibacterial against *Escherichia coli*, acting as a bacteriostatic agent able to influence several physiological processes [63]. A methodology was also developed to extract and encapsulate prodigiosin as a method focused on drug transport [75], and production methodologies are being optimized to favor the biosynthesis of this pigment [76]. One thing is clear, prodigiosin is a promising pigment in the implementation of new treatments and the development of new drugs.

## 8. Rhodopsins

Rhodopsin is the visual pigment expressed in the photoreceptor cells of retinal rods (Figure 2e) and is responsible for mediating vision with little light in vertebrates [64]. In the 1980s, it was discovered that not only higher organisms have this compound, given that Archaea such as *Halobacterium halobium* carried a second retinal pigment that worked in the microorganism as a sodium pump mediated by luminescence, which was called halorhodopsin [65]. In the following three decades, three new microbial rhodopsins were discovered in *H. halobium*, called bacteriorhodopsins, and these complemented the primary function of halorhodopsin, mediating the transmembrane ion flux using luminescence as the energy source [66,67,68,69]. In recent years, several rhodopsins have been described in different species of microorganisms capable of fulfilling multiple functions, from the mediation of the ion flux to phototaxis and photomobility (Table 1). Thus, rhodopsins are used as suitable models for understanding active membrane transport mechanisms and signaling sensors [70]. It has been discovered that bacteriorhodopsins can modulate cellular behavior via their luminescence-induced proton-exchange property; thus, bacteriorhodopsins could serve as material to directly influence the growth, metabolism, and differentiation of animal cell [71]. Simultaneously, this same approach is being used to reprogram human fibroblasts in neural cells to increase neural regeneration in the near future [72], with these investigations being some of the first biomedical applications of bacteriorhodopsins.

## 9. Pyocyanins and Pyoverdines

**Figure 4:**
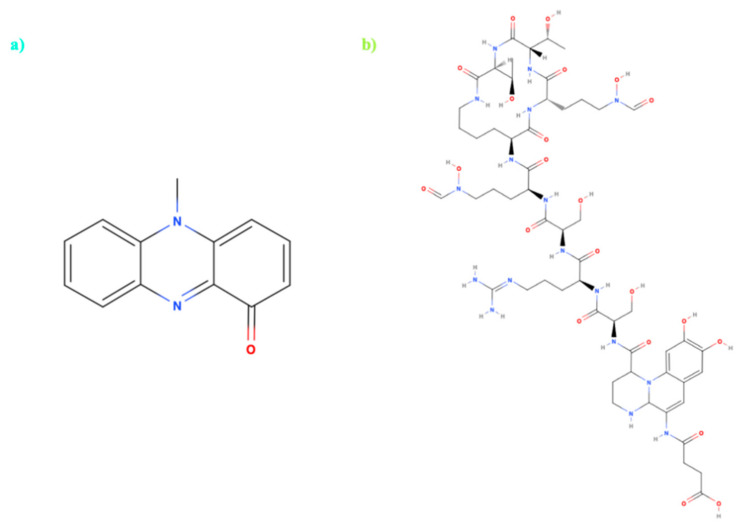
Molecular structures of (**a**) Pyocyanin and (**b**) Pyoverdin. Bacterial pigments and virulence factors are produced by the genus *Pseudomonas***.** (Structures made using MolView software) [74]).

The genus *Pseudomonas* can produce a variety of pigments as secondary metabolites, which play a very important role in the pathogenesis of these bacteria [73]. Pyocyanins and pyoverdines (Figure 4) are virulence factors produced by the strain *Pseudomonas aeruginosa* and are widely known for their capacity for iron uptake from the extracellular medium [77]. Pyocyanin, a greenish-blue color (Figure 4a), comes from the family of the phenazines and plays an important role in the metabolism of iron, being able to participate in reduction mechanisms and releasing iron from transferrins [78]. At the same time, pyocyanin has been shown to have numerous antagonistic effects, both in vivo and in vitro, due mainly to the pathogenic relation between *P. aeruginosa* and their host organism involving cell damage as the result of pro-inflammatory effects and the release of free radicals [79]. On the other hand, pyoverdine (Figure 4b) is a siderophore of bioluminescent greenish-yellow with high affinity or Fe (III), able to sequester iron from the environment and remove it from the host’s iron transport proteins, transferrins, and lactoferrins [80,81]. Thus, pyoverdines fulfill a key role in the development of the pathogenesis of *P. aeruginosa,* contributing to the acquisition of iron in vivo, with significant amounts of pyoverdines being found in the sputum of patients with cystic fibrosis [82]. The study of these pigments has focused mainly on the development of techniques to inhibit the production of virulence factors (Table 1), in order to mitigate *P. aeruginosa* infections in patients hospitalized with immunodeficiencies, cystic fibrosis, or with high resistance to antibiotics, where the treatment of infections by this pathogenic strain is becoming increasingly complex [80]. A large number of the studies on both compounds concentrate on their activities as virulence factors. In fact, in the case of pyoverdines, there is no relevant data that confirm biological properties beyond their action as a siderophore. On the other hand, recent studies have begun to attribute pyocyanin with new biological activities, reporting them as a biological control agent with antibiotic properties as well as having cytotoxic activity in pancreatic cancer cell lines [83,84]. However, their potentially toxic properties remain and the possible applications of this compound continue to be questionable.

## 10. Future Prospects

At present, biological prospecting of secondary metabolites opens up a range of possibilities for the discovery and application of biological properties, many of which remain hidden. Concerning natural pigments, microorganisms have been shown to be a valuable niche for acquiring harmless pigments, with various biotechnological applications that can be easily produced and scaled. This is where, of all existing microorganisms, bacteria must be considered an inexhaustible source of new natural pigments, which have been shown throughout this review to have an endless number of beneficial properties that can be applied as biomedical treatments. In this regard, it is expected that the study and prospecting of new varieties of pigments will lead to new biotechnological applications, either in the development of new treatments, the production of new drugs, or in the implementation of a new range of natural products harmless to humans. Accordingly, it becomes imperative and highly relevant to promote the study and search for new natural pigment-producing strains as well as to expand studies of the pigments previously described to clarify possible biological activities that have not yet been described.

## 11. Conclusions

Based on what was recorded throughout this review, we assessed the main biological activities of the most relevant bacterial pigments to date and how these biological compounds can offer quick solutions to current problems, efficiently and harmlessly for both humans and the environment. The bacterial pigments previously described in this review have high potential as novel biological compounds with diverse therapeutic applications, but there may still be a long way to go, so it becomes imperative to do a more in-depth study of each of these pigments, based on their molecular aspects, behavior and biosynthesis pathways with the purpose of a future scaling up and massification of these pigments in the industry. Finally, the composition of these pigments can be highly variable, which results in a wide range of compounds able to offer unique biological properties possibly still waiting to be discovered.

## Figures and Tables

**Table 1:** List of all bacterial pigments reviewed, their biological activities, and reported genera of pigment producers.

Pigment	Color	Biological Activity	Reported Producers	References
**Violacein**	Purple	Antibiotic Antiparasitic Antiviral Antitumoral Anticancer	*Chromobacterium* *Janthinobacterium* *Alteromonas* *Collimonas* *Duganella* *Pseudoalteromonas* *Iodobacter*	[15,16,17,18,19,20,21,22]
**Indigoidine**	Indigo	Antioxidant Signaling Antibiotic	*Arthrobacter* *Erwinia* *Corynebacterium* *Clavibacter* *Vogesella* *Phaeobacter* *Photorhabdus* *Dickeya* *Streptomyces*	[7,23,24,25,26,27,28,29]
**Melanin**	Dark Brown	Photoprotection Antioxidant Anticancer	*Rhizobium* *Bacillus* *Pseudomonas* *Klebsiella* *Modestobacter* *Streptomyces*	[30,31,32,33,34,35,36,37,38,39,40,41]
**Carotenoids**	Red/Orange	Antibiotic Antioxidant Cytotoxic activity	*Erwinia* *Flavobacterium* *Brevibacterium* *Paracoccus* *Pantibacter*	[9,42,43,44,45,46,47,48,49,50]
**Prodigiosin**	Deep red	Biocontrol Antibiotic Algaecidal Anti-inflammatory Anticancer Antimalarial Antidiabetic Immune system modulator	*Serratia* *Janthinobacterium* *Streptomyces* *Vibrio* *Hahella* *Zooshikella* *Pseudoalteromonas*	[5,6,51,52,53,54,55]
**Rhodopsins**	Light Pink	Active transport Signaling Cell behavior modulator (cell reprogramming)	*Halobacterium (Archaea)* *Halloterrigena (Archaea)* *Halorubrum (Archaea)* *Natromonas (Archaea)* *Anabaena (Eubacteria)* *Gloeobacter (Eubacteria)* *Magnetospirillum (Eubacteria)* *𝛾-Proteobacteria (Eubacteria)*	[56,57,58,59,60,61,62,63,64]
**Pyoverdine**	Yellowish green	Bioluminescence Virulence factor Iron uptake	*Pseudomonas*	[65,66,67,68,69]
**Pyocyanin**	Greenish-blue	Virulence factor Iron uptake Cytotoxic activity Antibacterial activity	*Pseudomonas*	[65,66,67,68,69,70,71,72,73]
